# 

**Published:** 1905-06

**Authors:** 


*THE BRISTOL MEDICO-CHIRURGICAL SOCIETY.*

### CONTENTS.

| | PAGE |
|---|---|
| **On Some Symptoms of Cerebellar Tumours.** By J. MICHELL CLARKE, M.A., M.D. Cantab., F.R.C.P. ... ... ... | 97 |
| **The Causes and Treatment of Baldness.** By HENRY WALDO, M.D., M.R.C.P. ... | 107 |
| **Acne Vulgaris and its Treatment.** By W. KENNETH WILLS, M.A., M.B., B.C. Camb. ... ... ... ... ... ... ... | 113 |
| **The Progress of Pharmacy and Pharmaceutical Chemistry.** By OLIVER C. M. DAVIS, B.Sc. Lond. ... ... ... | 120 |
| **Cases of Joint Swellings simulating Gout and Rheumatism.** By R. LLEWELYN JONES, M.B. Lond. ... ... ... ... ... | 127 |
| **A New Scheme for Infant Milk Depôts.** By J. M. FORTESCUE-BRICKDALE, M.A., M.D. Oxon. ... ... ... ... ... ... | 132 |
| **Sprue.** By CHARLES BEGG, M.B., C.M. Edin. | 138 |
| **Inebriety and the So-called Cures.** By JAMES STEWART, B.A., F.R.C.P. Edin. | 144 |
| **Progress of the Medical Sciences:** | |
| **Medicine.** GEORGE PARKER, M.D. Cantab. | 148 |
| **Surgery.** CHAS. A. MORTON, F.R.C.S. Eng. | 153 |
| **Obstetrics.** W. C. SWAYNE, M.D. Lond. | 156 |
| **Reviews of Books** ... ... ... ... ... ... | 161 |
| **Editorial Notes** ... ... ... ... ... ... | 173 |
| **Notes on Preparations for the Sick** ... | 177 |
| **The Library of the Bristol Medico-Chirurgical Society** ... ... ... ... | 181 |
| **Meetings of Societies** ... ... ... ... ... | 184 |
| **Obituary Notices** ... ... ... ... ... ... | 189 |
| **Local Medical Notes** ... ... ... ... ... | 190 |

